# Magnetic Calcium Phosphate Cement for Hyperthermia Treatment of Bone Tumors

**DOI:** 10.3390/ma13163501

**Published:** 2020-08-08

**Authors:** Ethel Ibinabo Ruskin, Paritosh Perry Coomar, Prabaha Sikder, Sarit B. Bhaduri

**Affiliations:** 1 Department of Mechanical Industrial & Manufacturing Engineering, The University of Toledo, Toledo, OH 43606, USA; Ethel.Ruskin@rockets.utoledo.edu (E.I.R.); sarit.bhaduri@gmail.com (S.B.B.); 2 College of Literature, Sciences & Arts, University of Michigan, Ann Arbor, MI 48109, USA; pcoomar@umich.edu; 3 ENG-EEC Division, The National Science Foundation (NSF), Alexandria, VA 22314, USA

**Keywords:** magnetic calcium phosphate cement, magnetite, hyperthermia

## Abstract

This article reports, for the first time, the ‘proof-of-concept’ results on magnetic monetite (CaHPO_4_)-based calcium phosphate cements (CPCs) compositions developed for the hyperthermia treatment of bone tumors. Hyperthermia involves the heating of a tumor within a temperature range of 40–45 °C, inducing apoptosis in the tumor cells. This process holds promising potential in the field of cancer treatment and has been proven to be more effective than conventional therapeutics. Hence, we aimed to develop cement compositions that are capable of the hyperthermia treatment of bone tumors. To achieve that central goal, we incorporated iron oxide (Fe_3_O_4_), a ferromagnetic material, into monetite and hypothesized that, upon the application of a magnetic field, magnetite will generate heat and ablate the tumor cells near the implantation site. The results confirmed that an optimized content of magnetite incorporation in monetite can generate heat in the range of 40–45 °C upon the application of a magnetic field. Furthermore, the compositions were bioactive and cytocompatible with an osteoblastic cell line.

## 1. Introduction

The major theme of this paper is to develop a monetite (dicalcium phosphate anhydrous, DCPA CaHPO_4_)-based calcium phosphate cement (CPC) composition capable of generating heat in the range of 40–45 °C to remove malignant bone tumors. The ultimate product of this effort is a self-setting iron oxide (Fe_3_O_4_)-ceramic composite that is capable of generating sufficient heat to kill cancer cells, while also preserving its bioactivity and biocompatibility with healthy bone cells. In the following, we describe the background and the fundamentals of developing this effective therapeutic in the field of bone cancer treatment.

It is well known that cancer cells have a high tendency to spread from the originating source (primary tumors) to other sites in the body (to form secondary tumors) through metastasis. Unfortunately, bone is a major site of metastasized cells, with 80% of them originating from malignant tumor cells of the breast and pancreas [1]. Nevertheless, primary bone tumors, referred to as sarcoma, constitute a deadly yet uncommon group of cancers [2]. The conventional treatment methods for treating such bone tumors are surgery, radiation therapy, and chemotherapy. However, these methods present shortcomings. For instance, surgery involves high mortality risks, post-operative distress, and high chances of a recurrence of the tumors. Radiation therapy may often be ineffective on tumor cells, and an increased dosage might cause necrosis of the surrounding healthy cells and damage of epithelial tissues. Chemotherapy requires a strict diagnosis and prognosis and has significant side effects on the post-treatment physical and mental states of a patient. On the contrary, hyperthermia treatment of tumors does not involve any of the above-mentioned shortcomings. The treatment involves a gradual increase of temperature in the target area, which stimulates the immune responses and helps in transforming the tumor cells into an anaerobic metabolic scheme, thus resulting in apoptosis [3]. Heat can be generated by ultrasounds, microwaves, radiowaves, or by introducing magnetic particles at the tumor site. The latter technique is known as ‘magnetic hyperthermia’ and works through the concept of heat generation by a magnetic material when an external alternating magnetic field is induced. Jose et al. recently reviewed the outstanding potential of applying magnetic nanoparticles for the hyperthermia treatment of cancer [4]. A key requirement is that the magnetic material must be biocompatible. Iron-oxide or magnetite (Fe_3_O_4_) has been proven to be biocompatible, in addition to its favorable ferromagnetic properties [5]. Furthermore, incorporating magnetite with orthopedic cements would be a promising therapeutic for bone tumors. While the magnetic component helps in generating heat in situ to kill tumor cells, the bone cement material can provide the scaffolding properties that are required for the regeneration of new healthy bone at the cancer site.

Following this strategy, Takagemi et al. incorporated magnetite into Polymethylmethacrylate (PMMA) and used the compositions for hyperthermia treatment [6]. The study revealed that the heat generation was dependent on the magnetite content and the intensity of the applied magnetic field. However, PMMA is not biodegradable and requires a second surgery to be removed, a major therapeutic drawback. In comparison, CPC compositions are biodegradable, in addition to their favorable biocompatibility. Xu et al. developed an optimized composition and quantity of injectable magnetite-incorporated β-tricalcium phosphate (β-TCP)-based CPC composition and employed an alternating magnetic field to successfully ablate liver tumors [7]. Furthermore, when the composition was injected into the center of the tumors in vivo, they were completely ablated after 180 secs of induction heating. Similarly, Yan et al. incorporated 10 wt.% iron oxide/graphene oxide nanocomposites into α-tricalcium phosphate (α-TCP)/calcium sulfate to develop the most stable bone cement, with an excellent biocompatibility and magnetothermal performance [8]. Recently, Xia et al. incorporated superparamagnetic iron oxide nanoparticles into a tetracalcium phosphate (TTCP, Ca_4_(PO_4_)_2_O) and dicalcium phosphate anhydrous (DCPA, CaHPO_4_)-based CPC. Under the application of a static magnetic field, the compositions exhibited a noteworthy enhancement of osteogenic differentiation in vitro (with human dental pulp stem cells) and active osteogenesis in vivo (in rat mandible defect models) [9]. In a follow-up study, the group confirmed that the enhancement in the osteogenic behavior was likely driven by the magnetic CPCs via the WNT/β-catenin signaling pathway [10]. Thus, it was confirmed that iron oxide, besides being able to generate heat in situ, possesses a favorable osteogenic biocompatibility as well. 

Understanding the potential of iron oxide as a therapeutic biomaterial, in the present study, we aimed to explore the heat generation and biocompatibility aspects of CPC compositions containing magnetite. One of the prime novelties of this study is that the CPC composition is monetite-based. Monetite is the latest innovation in CPCs, and over the last few years, our group has made a sustained research effort to develop monetite-based orthopedic cements [11,12,13,14,15]. Importantly, our synthesis protocol involves a low-temperature, rapid, simple microwave irradiation technique, which helps in developing non-exothermic monetite CPCs, a beneficial advantage in the arena of orthopedic cements [13,16]. Furthermore, the developed compositions are injectable, self-setting, biocompatible, and favorably biodegradable. Thus, the goal of this study was to provide ‘proof-of-concept’ results of novel compositions of monetite CPCs incorporated with iron oxide. The cements were explored on the grounds of their heat generation capability for hyperthermia treatment, and their biocompatibility properties for enhancing the osteogenic behavior. 

## 2. Materials and Methods

### 2.1. Magnetic CPC Preparation 

Monetite (CaHPO_4_)-based CPC was developed following a detailed procedure described by Koju et al. and Zhou et al. [12,13]. Briefly, the aqueous setting solution was added to a powder mixture containing calcium hydroxide (Ca(OH)_2_ Fischer Scientific, Waltham, MA, USA) and magnesium hydroxide (Mg(OH)_2,_ Fischer Scientific, Waltham, MA, USA) and mixed. Subsequently, the resultant mass was microwave-irradiated until it resulted in a rock-like material that was crushed to make fine CPC powder. As mentioned in Table 1, varying cement compositions were developed by combining powder mixtures with an aqueous solution. The powder mixture comprised CPC powder, magnesium oxide (MgO Millipore Sigma, St. Louis, MO, USA), sodium phosphate dibasic (Na_2_H_2_PO_4_, Fischer Scientific, Waltham, MA, USA), and different concentrations of magnetite powder. The aqueous solution comprised a clear blend of Na_2_H_2_PO_4_ in 2.5 wt.% colloidal silica. The liquid-to-powder ratio was always maintained at 0.35 mL/g. After mixing the powder mixture and the liquid, they were put in molds to form pellets. The compositions were referred to as MCPCs-xFe, which means magnetic calcium phosphate cements, with *x* indicating the varying weight content of magnetite incorporation, as shown in Table 1. 

### 2.2. Self-Setting Properties and Physical Characterization 

The initial and final setting times of various MCPCs were determined using the Gillmore needle method (ASTM C266-89). The setting times provide data relating to the workability of the cement with the initial time required for the cement to fully withstand a Gilmore needle with a tip diameter of 2.12 mm and weight of 113.4 g without showing any visible indentation of a depth greater than 1 mm on the surface of the newly formed cement pellet. The final setting time was measured at the time when the cement showed no indentation greater than 1 mm; there was no visible indentation on the surface of the cement when a Gilmore needle with a tip diameter of 1.06 mm and weight of 435.6 g was placed on the cement. The time was recorded at intervals of 15 s, with the needle depressed on the cement at these intervals for the initial and final setting time. 

Phase compositions and functional group analyses were detected using X-Ray Diffraction (XRD, Ultima III, Rigaku, USA) with monochromated Cu Kα radiation (44 kV, 40 mA), with a focused beam mode over a 2θ range of 10–60°. The step width and count time were fixed to be 0.05° and 8 s during the analysis. A scanning electron microscope (SEM, S-4800, Hitachi, Japan) was used to study the surface morphology of various MCPCs. 

### 2.3. Magnetic Characterization

The magnetic property of MCPCs was performed by placing the cylindrical specimens (Ă7 × 6 mm) in a beaker containing colloidal silica. The set-up was placed in a zero-voltage switching induction coil (7.5 × 2.8 cm^2^) connected to a DC supply with a voltage range of 5–12 V. The induction heating generated from the cements was recorded using an infrared thermometer.

### 2.4. Bioactivity 

To ensure the bioactivity of the MCPCs, specimens (Ă7 × 6 mm) were immersed in 1.5× simulated body fluid (1.5-SBF) for three, seven, or 14 days in a water bath incubator that maintained 37 ± 0.5 °C. The ionic compositions of 1.5 SBF mimic the concentration of human blood plasma [17]. At the end of the test period, they were retrieved, completely dried, and analyzed using SEM. 

### 2.5. Cytocompatibility

For the cytocompatibility analyses of the specimens, model osteoblastic cell line–mouse calvarial pre-osteoblasts designated as MC3T3-E1 (ATCC CRL-2593, Manassas, VA, USA) were used. Specimens were autoclaved and cooled down to room temperature before every assay. All the assays were performed after the specimens were completely set (after the final setting time). Approximately 2.3 × 10^4^ cells were seeded directly on different MCPC specimens and cultured in complete MEM-α at 37 °C with 5% CO_2_ flow for seven days. Wells with no samples and only cells served as controls. At that specific time frame, well plates were retrieved from the incubator and treated with 500 µL thiazolyl blue tetrazolium bromide (MTT, Sigma-Aldrich, St. Louis, MO, USA) for 4 h at 37 °C with 5% CO_2_ flow. After 4 h, dimethyl sulfoxide (DMSO, Sigma Aldrich, St. Louis, MO, USA) was used to dissolve the formazan during a short 10 min incubation. Subsequently, OD_540_ readings were recorded by spectrophotometer and compared. 

### 2.6. Statistical Analysis

Tests were carried out in triplicates. A one-way Analysis of variance (ANOVA) with a Tukey’s test was conducted, and ρ < 0.05 was considered significant. 

## 3. Results and Discussion 

### 3.1. Self-Setting Properties and Material Characterization 

The setting times and handling conditions for various MCPCs are tabulated in Table 2. The setting times of the compositions are acceptable for cement workability and are in good accordance with previous results [12]. CPCs with initial setting times in the range of 3–8 min and final setting times of less than 15 min satisfy the requirement for cement application in clinical use [18]. It provides sufficient time for orthopedic surgeons to prepare the CPC by mixing the powder phase with the liquid and to apply them at the desired site. However, the MCPC-40Fe composition exhibits much shorter initial setting and final setting times, thus compromising the favorable handling conditions for molding or injectability. This might be due to the faster crystal growth at the reinforcement-matrix interface resulting from the presence of Ca binding groups or higher siloxane groups. 

**Figure 1:**
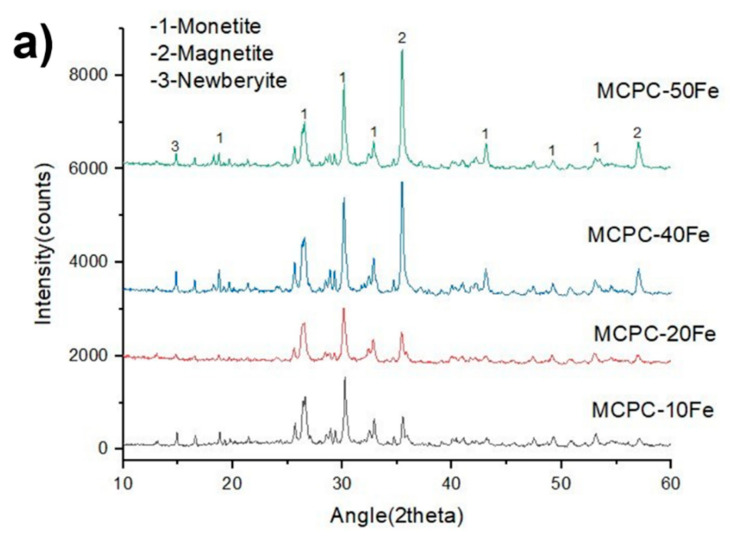

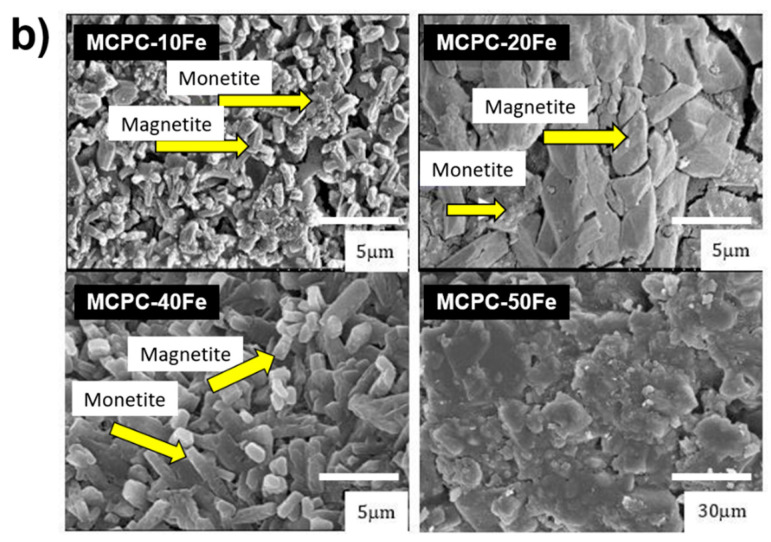
(**a**) X-ray diffraction analysis and (**b**) Scanning electron microscopy (SEM) images of MCPC-10 Fe, MCPC-20, Fe MCPC-40, and Fe MCPC-50 Fe. MCPC indicates magnetic calcium phosphate cement, and Fe indicates magnetite incorporation.

The XRD phase analyses in Figure 1a show the prominent diffraction peaks of two major phases: monetite (PDF# 97-000-0918) and magnetite (PDF#97-003-156). In lower concentrations of magnetite, i.e., in MCPC-10 and MCPC-20Fe compositions, the diffraction peaks of monetite are high, but with a higher incorporation content of magnetite (MCPC-40Fe and MCPC-50Fe), the diffraction intensity of the peaks corresponding to magnetite is significantly higher than for monetite. Furthermore, the well-defined nature of the peaks indicates the crystalline nature of both phases. Newberyite (Mg(HPO_4_).3H_2_O, PDF #97-003-1281) is also seen as being present in minute quantities in all the compositions due to usage of Mg(OH)_2_ while preparing the cement. 

Figure 1b shows the SEM micrographs, which display the surface morphologies of different MCPC compositions. MCPC-10Fe display irregular, block-like structures clustered on the cement surface. This structural morphology can be correlated to the tetrahedral lattice structure of magnetite [19]. In the case of MCPC-20Fe, the microstructures reveal a more compact, planar, rock-like surface. The dome-shaped magnetite particles are seen to be clustered on the cement surface. Importantly, this morphological feature is a distinct characteristic of magnetite, consisting of a face-centered cubic pattern resulting from its lattice structure [20]. The increase in magnetite incorporation resulted in significant morphological differences. The block-like particles appeared flatter and more congregated in MCPC-40Fe than in MCPC-10Fe. MCPC-50Fe revealed a completely different microstructure than all the other compositions. The crystals were observed to be interwoven, which resulted in a unified microstructure with no specific grain boundaries. This could be due to the presence of the high magnetite content, which tends to coagulate together [21]. Moreover, the monetite microstructure was sparsely visible on the cement surface. The high content of magnetite might have possibly acted as a suppressant to the initial formation of the calcium phosphate phase, thereby resulting in the presence of a minimal monetite microstructure. 

### 3.2. Heat Generation by MCPCs 

**Figure 2:**
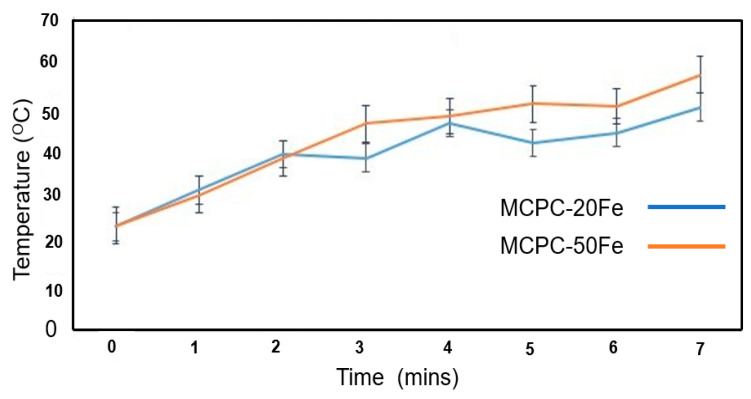
Heat generation profile of MCPC with different concentrations of magnetite. MCPC indicates magnetic calcium phosphate cement, and Fe indicates magnetite incorporation.

Figure 2 shows the heat generation profile of the MCPC compositions over 7 min. At the onset of the application of the electromagnetic field, the initial temperatures for both the cement compositions gradually increase. Please note that the heat generated by MCPC-10Fe is very similar to MCPC-20Fe and that the heat generated by MCPC-40Fe is very similar to MCPC-50Fe. In order to draw a better comparison and note the difference, we chose to present the results of MCPC-20Fe and MCPC-50Fe. During the first 2 min, no notable difference in heat generation is observed between MCPC-20Fe and MCPC-50Fe. Interestingly, after 2 min, the temperature of the MCPC-20Fe drops noticeably over a minute before rising back again. This pattern is also repeated at the 4th minute. On the contrary, the heat generated by MCPC-50Fe consistently increases over time and reaches a maximum of 55 °C, which might be harmful to healthy cells. Conversely, despite a non-uniform heat generation profile in the case of MCPC-20Fe, the localized temperature lies in the range of 40–45 °C, which is enough to kill the tumor cells without destroying healthy cells [22,23]. In a recent study, Yan et al. confirmed that the temperature range of 43−45 °C was sufficient to kill tumor cells while having a less damaging effect on stem cells [8]. In another study, 0.36 g of 10 wt.% magnetite-incorporated CPC compositions generated a temperature of approximately 46 °C and successfully ablated 1.96 ± 0.19 cm^3^ of tumors [7]. Heat is generated due to the excitation and random movement of the magnetite particles under the application of an electromagnetic field. The extent of heat generation can be controlled by varying the amount of incorporated magnetic component and/or by configuring the parameters of the applied electromagnetic field. In this study, we varied the amount of magnetic component to optimize the heat generation. The result indicates that the MCPC-20Fe composition is capable of generating an optimized heat for the hyperthermia treatment of bone tumors.

### 3.3. Bioactivity 

**Figure 3:**
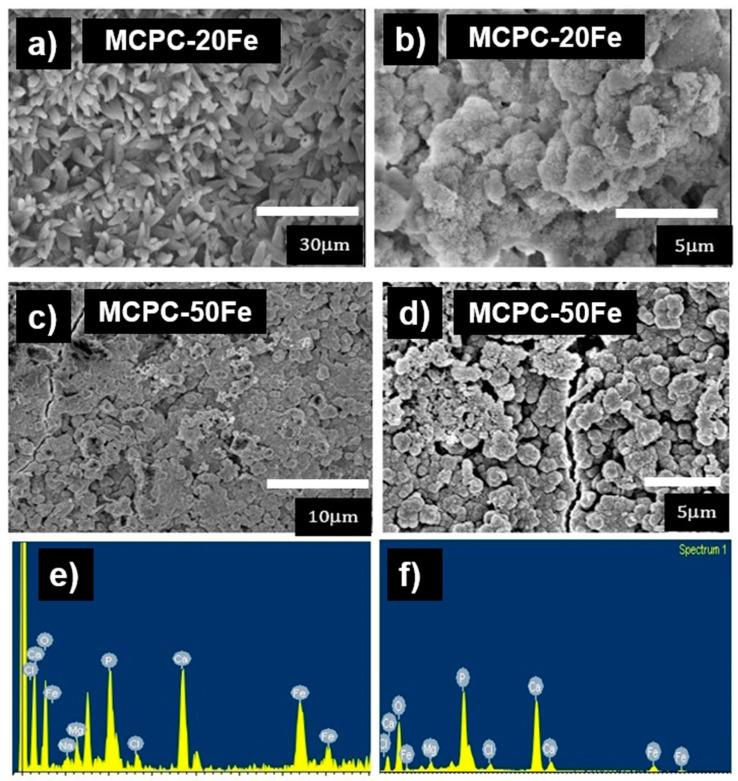
SEM images and EDS spectrum of MCPC with different concentrations of magnetite after a seven-day immersion in Simulated Body Fluid (SBF). (**a**) Low magnification micrographs of MCPC-20Fe show the formation of elongated petal-like apatite crystals. (**b**) High magnification of (**a**) shows the globular structure and the nanoflake-like nature of the apatite particles. (**c**) Low magnification micrographs of MCPC-50Fe indicate the formation of a dense layer of apatite, totally covering the cement surface. (**d**) High magnification of (**c**) reveals the globular structures of apatite. (**e**) EDS signals of MCPC-20Fe. (**f**) EDS signals of MCPC-50Fe. MCPC indicates magnetic calcium phosphate cement, and Fe indicates magnetite incorporation.

Figure 3a–f shows the SEM micrographs and EDS analysis of the cements after a seven-day immersion in SBF. The low magnification micrographs of the MCPC-20Fe surface (Figure 3a) display the presence of elongated petal-like crystals with tiny orifices at the tip, indicating the formation of mineral apatite [24]. The higher magnification micrograph in Figure 3b confirms the formation of a globular structure with a nanoflake-like morphology, a signature aspect of bone-like apatite formed due to SBF immersion [25]. Furthermore, the Ca/P ratio of the globular structures is calculated to be 1.5 from the EDS analysis, as shown in Figure 3e. This confirms the formation of calcium-deficient apatite. Additionally, the analysis picked up high intensity EDS signals of Ca and P. In the case of MCPC-50Fe, the low magnification micrograph, as shown in Figure 3c, indicates the formation of a dense layer of apatite that totally covers the cement surface. This could be due to the presence of a higher magnetite or iron content, which can be responsible for enhancing the bioactivity of the cement [5,26]. The higher magnification micrographs in Figure 3d confirm the formation of bone-like apatite by revealing similar globular structures. Figure 3f shows the EDS signals picked up from MCPC-50Fe after SBF immersion. Interestingly, EDS picked up low signals of Fe and a higher concentration of Ca and P (Ca/P ratio 1.6) due to the thick layer of apatite, which concealed the original surface of the cement. Thus, the SBF immersion study confirms that the MCPC cement compositions are capable of forming apatite, the intermediate layer required for osseointegration (binding with bone), thus highlighting their favorable bioactive nature. 

### 3.4. In Vitro Cytocompatibility 

**Figure 4:**
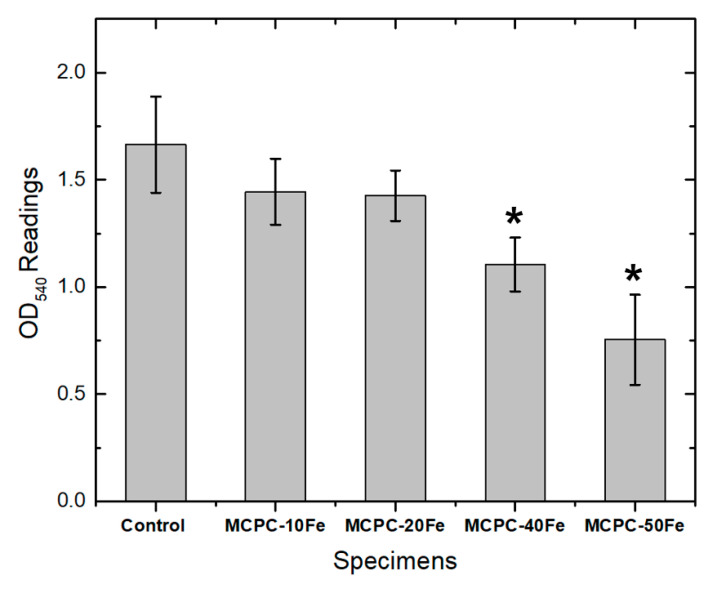
OD_540_ readings indicating the cell viability results of MC3T3 pre-osteoblasts cultured on MCPC with different concentrations of magnetite. MCPC indicates magnetic calcium phosphate cement, and Fe indicates magnetite incorporation.

Preliminary cytocompatibility studies are important before analyzing the biomaterials in vivo. In an MTT assay, OD readings represent a direct correlation with the cell viability [12,27]. Figure 4 shows the viability of MC3T3 pre-osteoblast cells cultured on different MCPC compositions. MCPC-10Fe and MCPC-20Fe exhibit comparable OD readings to the control, indicating that the compositions are cytocompatible with pre-osteoblast cells. However, with an increasing magnetite concentration, the OD readings decrease and are statistically significant with respect to the control. This could be due to an increased degradation rate of the compositions with a higher magnetite content, thus resulting in an excess release of the iron oxide particles that can cause cytotoxicity [5,28]. Thus, it is preferable to use compositions with a lower content of magnetite, like MCPC-20Fe, to preserve the cytocompatible nature of the cements. 

## 4. Conclusions

The present study presents “proof-of-concept” results with noteworthy implications. We aimed to develop an optimized composition of magnetic bone cement and hypothesized that the heat generated upon the application of an external stimulus would be sufficient to kill tumor cells. The results indicate that MCPC-20Fe is the most appropriate composition. It exhibited acceptable initial and final setting times, indicating that the cements can be easily molded into various shapes and injected if needed. The material characterization results indicated the presence of crystalline monetite and magnetite in the composition. The SBF immersion studies confirmed the favorable bioactivity of MCPC-20Fe, thus highlighting their capability to osseointegrate in vivo. Most importantly, this cement composition generated heat in the range of 40–45 °C when an electromagnetic field was applied. The generated heat is enough to kill the tumor cells without destroying healthy cells. The in vitro studies further confirmed that the composition was biocompatible with pre-osteoblast cells. However, the present study is just a ‘proof-of-concept’ and has limitations. Future studies should be performed to analyze the therapeutic potential of the developed bone cements. Indeed, it would be worthwhile to conduct comprehensive studies that would explore the hyperthermia treatment potential of the bone cement compositions both in vitro and in vivo using proper models. 

## Figures and Tables

**Table 1:** Different compositions used for developing magnetic cements. MCPC indicates magnetic calcium phosphate cement, and Fe indicates magnetite incorporation.

Specimen Name	CPC/ Monetite (g)	Magnetite (g)	MgO (g)	Na_2_H_2_PO_4_ (g)	Colloidal Silica Blend (mL)
MCPC-10Fe	5	0.5	0.08	0.04	1.79
MCPC-20Fe	5	1	0.08	0.04	1.89
MCPC-40Fe	5	2.0	0.08	0.06	2.24
MCPC-50Fe	5	2.5	0.08	0.06	2.40

**Table 2:** Setting time of the different compositions of magnetic cements.

Cement Compositions	Initial Setting Time (min)	Final Setting Time (min)	Handling
MCPC-10Fe	5.23 ± 1.3	14.5 ± 4.5	A sticky consistency that stuck to gloves during cement preparation. This allowed for a good injectability.
MCPC-20Fe	5.72 ± 1.4	14.5 ± 4.5	Had a stickier consistency as the iron content in the cement increased. Cement was difficult to wash off.
MCPC-40Fe	4.40 ± 1.4	6.04 ± 0.15 *	Hardened at a faster rate compared to the other MCPC. Was difficult to work with.
MCPC-50Fe	7.75 ± 0.43 *	14.4 ± 1.8	Cement had a good workability and handling.

MCPC indicates magnetic calcium phosphate cement, and Fe indicates magnetite incorporation. * means statistically significant with respect to all the other samples.
